# Early menarche and childhood adversities in a nationally representative sample

**DOI:** 10.1186/1687-9856-2014-14

**Published:** 2014-07-15

**Authors:** Kimberly L Henrichs, Heather L McCauley, Elizabeth Miller, Dennis M Styne, Naomi Saito, Joshua Breslau

**Affiliations:** 1 University of Wisconsin School of Medicine and Public Health, 600 Highland Avenue, Madison, WI 53792-4108, USA; 2 University of Pittsburgh School of Medicine, 3420 Fifth Avenue, Pittsburgh, PA 15213, USA; 3 Children’s Hospital of Pittsburgh, University of Pittsburgh Medical Center, 3420 Fifth Avenue, Pittsburgh, PA 15213, USA; 4 University of California Davis, 2516 Stockton Blvd, Sacramento, CA 95817, USA; 5 University of California Davis, 1616 DaVinci Court, Davis, CA 95618, USA; 6 RAND Corporation, 4570 Fifth Avenue, Suite 600, Pittsburgh, PA 15213, USA

## Abstract

**Background:**

Epidemiological evidence suggests that early menarche, defined as onset of menses at age 11 or earlier, has increased in prevalence in recent birth cohorts and is associated with multiple poor medical and mental health outcomes in adulthood. There is evidence that childhood adversities occurring prior to menarche contribute to early menarche.

**Methods:**

Data collected in face-to-face interviews with a nationally representative sample of women age 18 and over (N = 3288), as part of the National Comorbidity Survey-Replication, were analyzed. Associations between pre-menarchal childhood adversities and menarche at age 11 or earlier were estimated in discrete time survival models with statistical adjustment for age at interview, ethnicity, and body mass index. Adversities investigated included physical abuse, sexual abuse, neglect, biological father absence from the home, other parent loss, parent mental illness, parent substance abuse, parent criminality, inter-parental violence, serious physical illness in childhood, and family economic adversity.

**Results:**

Mean age at menarche varied across decadal birth cohorts (χ^2^₍₄₎ = 21.41, p < .001) ranging from a high of 12.9 years in the oldest cohort (age 59 or older at the time of interview) to a low of 12.4 in the second youngest cohort (age 28-37). Childhood adversities were also more common in younger than older cohorts. Of the 11 childhood adversities, 5 were associated with menarche at age 11 or earlier, with OR of 1.3 or greater. Each of these five adversities is associated with a 26% increase in the odds of early menarche (OR = 1.26, 95% CI 1.14-1.39). The relationship between childhood sexual abuse and early menarche was sustained after adjustment for co-occurring adversities. (OR = 1.77, 95% CI 1.21-2.6).

**Conclusions:**

Evidence from this study is consistent with hypothesized physiological effects of early childhood family environment on endocrine development. Childhood sexual abuse is the adversity most strongly associated with early menarche. However, because of the complex way that childhood adversities cluster within families, the more generalized influence of highly dysfunctional family environments cannot be ruled out.

## Background

Among industrialized nations, and the United States in particular, the average age of menarche has decreased over the past century [1-3]. This trend is of public health concern as early menarche, commonly defined as onset of menses before age 12, is associated with multiple poor health outcomes in adults from increased cardiovascular and metabolic diseases to breast cancer and all-cause mortality [4-6]. Early menarche has also been linked to increased health risk behaviors, earlier sexual debut, and depression [7-9].

Early menarche is likely the result of multiple influences on early endocrine development. Epidemiological studies have found that early menarche is associated with multiple inter-related social and environmental factors, including nutrition and obesity [10-12], genetic factors [13,14], and general health status [15], with differences noted by race/ethnicity and familial socioeconomic status [16-18]. Among social factors with potential influence on early menarche, a growing body of literature points to exposure to childhood adversities as a risk factor for early menarche, including childhood sexual abuse [9,19-34]. The adverse impacts of biological father absence and the presence of non-related males in the home have also been suggested, though the evidence remains mixed [26,35-41]. To the best of our knowledge, studies to date have not considered the effects of a broader range of related childhood adversities on menarche. As adversities tend to cluster together, identifying specific adversities associated with early menarche could help identify particular environmental influences on endocrine pathways, should they exist. Elucidating the types of adversities associated with early onset of menarche is critical not only for guiding research on the impact of stressors on neuroendocrine development but also informing the design of targeted interventions for children exposed to specific adversities and potentially mitigating the impact of early pubertal development. This study assesses the joint predictive effects on age of menarche of a broad range of childhood adversities, including absence of biological father in the home and childhood sexual abuse, utilizing a large nationally representative sample from the United States, the National Comorbidity Survey Replication (NCS-R).

## Methods

### Sample

The NCS-R is a multi-stage clustered area probability survey (respondents selected into the sample were clustered within selected geographic units [42]) representative of the adult (ages 18+), non-institutionalized, civilian, English speaking population of the continental US focused on the prevalence and correlates of mental disorders [43,44]. Non-clinician interviewers conducted computer assisted face-to-face structured interviews between February 2001 and April 2003. The NCS-R interview schedule was the version of the World Health Organization (WHO) Composite International Diagnostic Interview (CIDI) developed for the WHO World Mental Health Survey Initiative [38,44,45]. The Human Subjects Committees of both Harvard Medical School and the University of Michigan approved the NCS-R recruitment, consent and field procedures. All NCS-R data and documentation are publically available at http://www.icpsr.umich.edu/CPES.

All respondents (N = 8098) completed a Part I core diagnostic interview. To reduce respondent burden, a subsample (selected with known probabilities) received a Part II interview, which included assessments of risk factors, consequences, services and other correlates of the core disorders. The Part II sample included all Part I respondents age 25 or younger, all Part I respondents meeting initial criteria for a mental disorder, and a random sample of Part I respondents over the age of 25 who did not meet criteria for a mental disorder. Sample weights accounted for differential probability of selection and non-response. Additional weights helped match demographic characteristics of the sample to those of the target population [43,44]. Of the 3,310 women completing the Part II interview, 22 provided no information on age at menarche and were excluded from the analysis, leaving a sample of 3288 women.

### Measures

#### *Early menarche*

Age at menarche was the self-reported age at first menstrual period. Early menarche was defined as occurrence of menarche at 11 years of age or less, consistent with values equal to or less than the 25^th^ percentile for age as published from national estimates of menarche timing for non-Hispanic White U.S. girls in the National Health and Nutrition Examination Survey III (1988-1994) [46]. Missing values for age of menarche were imputed for 40 participants who did not recall their exact age using information from questions about whether their first period occurred about the same time as, earlier than, or much earlier than their peers. Ages were imputed using the average age intervals associated with these categories among the large majority of respondents who provided their exact age at menarche.

#### *Childhood adversities*

Eleven childhood adversities were included in the analyses -- (1) physical abuse, (2) sexual abuse, (3) neglect, (4) biological father absence from the home, (5) other parent loss, (6) parent mental illness, (7) parent substance abuse, (8) parent criminality, (9) inter-parental violence, (10) serious physical illness in childhood, and (11) family economic adversity. Physical abuse and inter-parental violence were coded as moderate physical violence towards the respondent by the parent or adult caregiver or between parents or adult caregivers, respectively, using the revised Conflict Tactics Scale [47]. Sexual abuse was assessed with questions developed for the baseline National Comorbidity Survey about rape, sexual assault and molestation [48]. Neglect was assessed with a five-item scale developed for child welfare studies [49].

Absence of biological father was defined using a series of questions about respondents’ family structure during childhood. Respondents were first asked whether they had lived their entire childhood up to age 16 with both biological parents in their household. Respondents who answered ‘no’ to this question were considered to have had an absent biological father if they had more than one adult male living in their household for at least six months during childhood, the male head of household for most of their childhood was not their biological father, their biological father died prior to their age at menarche, they lived in foster care or with an adoptive family, or they had no male head of household for most of their childhood.

Parent mental illness and substance use disorders were assessed using the family history research diagnostic criteria, for either male or female adult caregiver [50,51]. Parental criminality was assessed through questions about whether the respondent’s parents were involved in criminal activities, arrested or sent to prison. Economic adversity was defined as having received welfare or not having a working parent as head of household. Information on the timing of parental death, divorce, separation, serious physical illness, inter-parental violence and sexual abuse was used to determine whether these childhood adversities began before the initiation of menstruation.

#### *Additional covariates*

Body mass index at time of interview was calculated from self-reported height and weight. Z-scores for variation in an individual’s BMI (body mass index) from the mean of their birth cohort were calculated and used to adjust statistically for BMI, given the relative stability of BMI within cohorts over time [52,53]. Race-ethnic categories were defined as mutually exclusive categories based on self-report. Age groups were defined to produce five birth cohorts of approximately equal size.

### Statistical analysis

Prevalence of early menarche was calculated as the proportion of respondents who reported that their first menstrual period occurred at 11 years of age or younger. Discrete time survival models with time-varying covariates were used to estimate associations of childhood adversities with early menarche. Survival models were adjusted statistically for age, race/ethnicity and BMI. Data were arranged by person-year (from age 6 to age 11 or age of menarche, whichever came first) with time-varying indicators for each childhood adversity. The survival models include one observation per year of age starting with age 6. If a respondent reported an adversity occurring prior to age 6, then that adversity was coded as present at age 6 (and all subsequent ages). Associations between childhood adversities and early menarche were calculated as adjusted odds ratios. Confidence intervals and statistical tests were calculated using the Taylor series linearization method as implemented in the SUDAAN software package to account for the complex sample design of the NCS-R [54]. Statistical significance was assessed using two-sided .05 level tests.

## Results

### Sample characteristics and cohort variation in age at menarche and childhood adversities

The mean age of the sample was 45.7 years (se = 0.60). 72% were White, 13% non-Hispanic Black, 10% Hispanic, and 4% other race/ethnicity. Mean BMI was 27.

**Figure 1:**
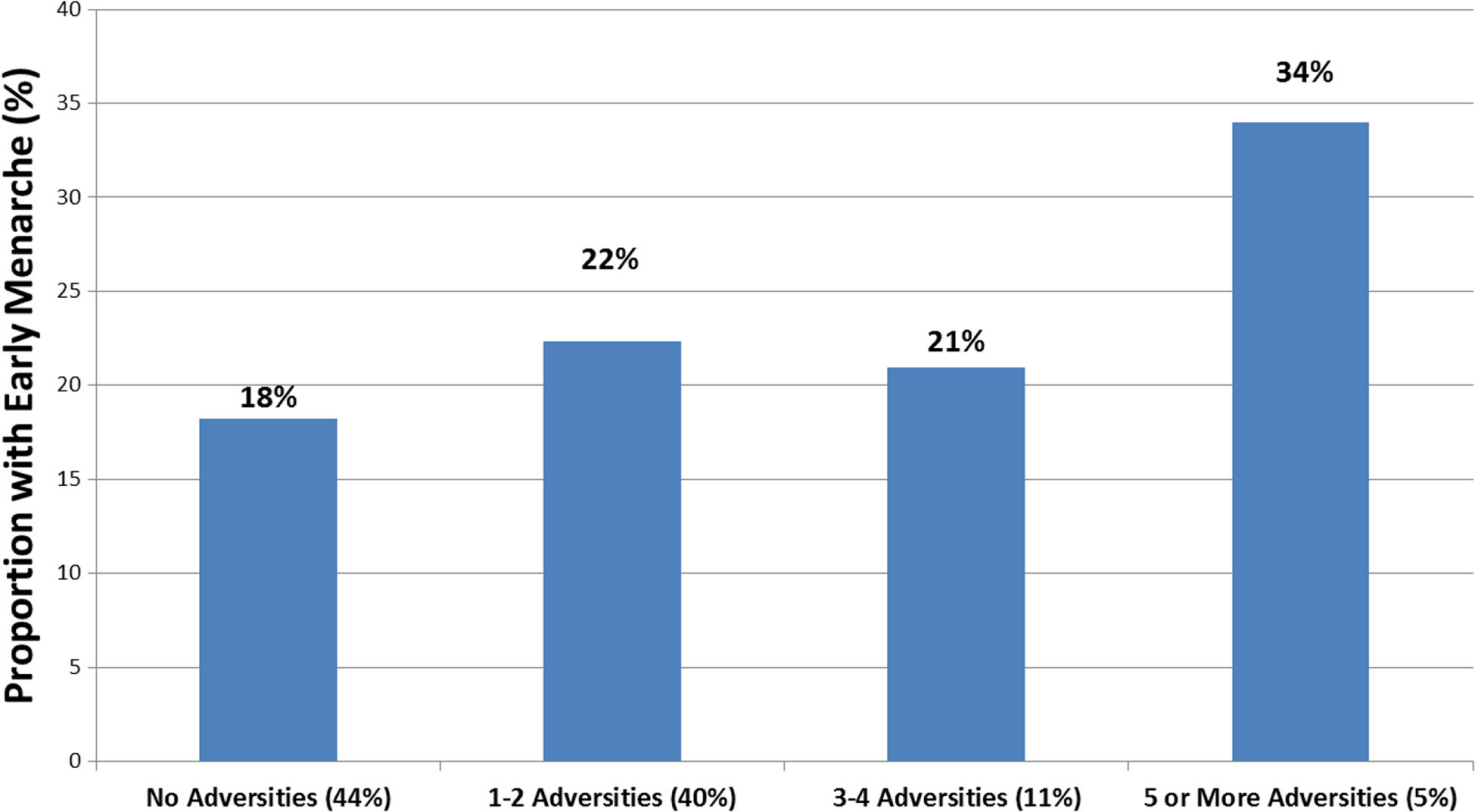
Early menarche by number of childhood adversities (Women age 18 and over, NCSR (N = 3,288).

The mean age at menarche was highest (12.9 years) in the oldest birth cohort, those age 59 and over at the time of interview, but the decline was not monotonic across birth cohorts (Table 1). Variation in the prevalence of childhood adversities across birth cohorts follows a similar pattern. Just over half (52.8%) of the eldest cohort reported no adversities in contrast to 38.2% of the youngest cohort (i.e. those ages 18-27). In particular, younger cohorts reported increased frequency of biological father absence (χ^2^ = 84.01, p <0.001), a childhood adversity theorized to have a particularly strong connection to early menarche. The proportion of girls not living with their biological fathers was more than twice as high in youngest versus oldest birth cohorts. Childhood sexual abuse was reported with greater frequency among the two younger cohorts (χ^2^ = 25.13, p < 0.001). The 28-37 year old cohort reported the highest prevalence of early menarche (24.9%, CI 21.3-28.8), the youngest mean age of menarche (12.4 years), the greatest exposure to adversities (66.5%), and the highest prevalence of multiple co-occurring adversities.Figure 1 shows the prevalence of early menarche across groups defined by the number of pre-menarchal childhood adversities. The prevalence of early menarche is close to 20% among people reporting no adversities, 1 or 2 adversities and 3 or 4 adversities. Elevated prevalence of early menarche is limited to the group with 5 or more adversities among whom it reaches 34%.

**Table 1:** Age of menarche, absence of bio-father, childhood sexual abuse (prior to menarche), and number of childhood adversities across birth cohorts

**Age**		**AOM**	**Early menarche**	**Not Lived with Bio-father**	**Childhood sexual abuse**	**Number ACE**				**Number ACE**
						**0**	**1,2**	**3,4**	**4 <**	
	**N**	**Mean (SD)**	**% (n)**	**% (n)**	**% (n)**	**% (n)**				**Mean (SD)**
18-27y/o	654	12.5 (1.57)	20.2 (158)	37.1 (243)	15.8 (133)	38.2 (221)	40.2 (266 )	15.4 (104)	6.2 (63)	1.4 (1.55)
28-37y/o	655	12.4 (1.31)	24.9 (163)	33.4 (206)	18.7 (144)	33.5 (210)	44.0 (286)	15.9 (99)	6.7 (60)	1.6 (1.50)
38-47y/o	697	12.7 (1.62)	20.4 (152)	21.1 (149)	14.8 (128)	47.9 (290)	40.0 (281)	8.1 (80)	4.2 (46)	1.0 (1.36)
48-58y/o	636	12.6 (1.52)	22.5 (146)	15.5 (112)	14.8 (118)	49.4 (276)	37.0 (249)	9.1 (73)	5.0 (38)	1.1 (1.42)
59-98y/o	646	12.9 (1.84)	17.7 (126)	16.2 (104)	7.3 (57)	52.8 (331)	38.6 (250)	7.6 (52)	0.9 (13)	0.8 (1.21)
Wald chi-square		χ^2^₍₄₎ = 21.41	χ^2^₍₄₎ = 9.43	χ^2^₍₄₎ = 84.01	χ^2^₍₄₎ = 25.13	χ^2^₍₁₂₎ = 123.62				χ^2^₍₄₎ = 46.46
P-value		<0.001	0.051	<0.001	<0.001	< 0.001				<0.001

Birth cohorts divided by decade of birth; percentages are weighted.

AOM = age of menarche.

ACE = adverse childhood experiences.

### Associations of pre-menarchal childhood adversities with early menarche

An initial set of models was specified to identify the adversities most strongly associated with early menarche, adjusting for age, ethnicity and BMI, but without adjustment for co-occurring adversities (Table 2). Of the 11 childhood adversities tested, only 4 were significantly associated with early menarche at the p = .05 level. Odds ratios for these four adversities (physical abuse, sexual abuse, parental mental illness and family violence) ranged from 1.36 to 2.19. The odds ratio associated with parental substance use was of similar magnitude, 1.3, but did not reach statistical significance (95% CI 0.91-1.91). Absence of the biological father from the home was not associated with early menarche (OR = 1.08 95% CI 0.86- 1.36).

**Table 2:** Associations of individual adverse childhood experiences with early menarche

	**Odds ratio**	**95% CI**	**Wald ChiSq**
Neglect	1.03	(0.63, 1.67)	0.01
Parent criminality	1.05	(0.77, 1.43)	0.10
Bio-father absent	1.08	(0.86, 1.36)	0.48
Physical illness	1.08	(0.71, 1.64)	0.13
Other parent loss	1.15	(0.63, 2.09)	0.23
Economic adversity	1.23	(0.88, 1.73)	1.53
Parent substance abuse	1.30	(0.91, 1.91)	2.15
Parent mental illness	1.36	(1.08, 1.71)	7.32**
Family violence	1.40	(1.03, 1.89)	4.97***
Physical abuse	1.81	(1.32, 2.48)	14.59*
Sexual abuse	2.19	(1.54, 3.12)	20.22*

*p < 0.001. **p < 0.01. ***p < 0.05.

Each odds ratio estimated in a separate model with statistical adjustment for age and race/ethnicity.

The five childhood adversities most strongly associated with early menarche were entered into two models to determine the joint effects of co-occurring adversities on early menarche (Table 3). In the first model, a simple count of adversities was entered as a continuous variable, with statistical adjustment for age, ethnicity, and BMI. Each adversity was associated with a statistically significant 26% increase in the odds of early menarche. In the second model, to examine the relative impact of specific adversities on early menarche, all five adversities were entered as simultaneous predictors (main effects model), with no interactions among the adversities. All odds ratios in the second model exceeded 1, but only sexual abuse reached statistical significance (OR = 1.77, 95% CI 1.21-2.60). Tests of statistical interactions between co-occurring adversities, including tests for diffuse interactions, were not statistically significant. In addition, tests for statistical interactions between race/ethnicity and childhood adversities were not statistically significant.

**Table 3:** Adjusted associations of number of adversities and individual types of adversities with early menarche

		**Model 1**		**Model 2**	
		**Adjusted odds ratio**	**95% CI**	**Adjusted odds ratio**	**95% CI**
**Age (years)**	18-27	1		1	
	28-37	1.22	(0.90, 1.66)	1.23	(0.90, 1.67)
	38-47	0.94	(0.67, 1.32)	0.94	(0.67, 1.33)
	48-58	1.13	(0.84, 1.54)	1.14	(0.83, 1.56)
	59-98	0.97	(0.68, 1.39)	0.98	(0.68, 1.41)
**Ethnicity**	White	1		1	
	Hispanic	1.49	(1.02, 2.15)	1.47	(1.00, 2.15)
	Black	1.45	(0.94, 2.22)	1.44	(0.94, 2.21)
	Other	0.61	(0.33, 1.16)	0.61	(0.31, 1.20)
**BMI (standardized)**		1.22	(1.12, 1.33)	1.22	(1.12, 1.33)
**Number of adversities**		1.26	(1.14, 1.39)	--	--
**Type of adversities**	Parent substance abuse	--	--	1.06	(0.74, 1.50)
	Parent mental illness	--	--	1.18	(0.91, 1.55)
	Family violence	--	--	1.14	(0.87, 1.50)
	Physical abuse	--	--	1.35	(0.98, 1.88)
	Sexual abuse	--	--	1.77	(1.21, 2.60)

Model 1: Number (count) of adversities as a continuous predictor with statistical adjustment for age, ethnicity, and BMI (i.e. ORs in model 1 are adjusted for all variables in the first column).

Model 2: Main effects of the five childhood adversities with the strongest associations with early menarche with statistical adjustment for age, ethnicity, and BMI (i.e. ORs in model 2 are adjusted for all variables in the second column).

## Discussion

Childhood adversities predict earlier onset of menarche in this large national sample of women, after statistical adjustment for BMI and race/ethnicity. The prevalence of early menarche is elevated at high levels of co-occurring adversities, specifically in the 5% of the population with more than five adversities. Among respondents reporting between one and four adversities (51% of the sample), the prevalence of early menarche was not significantly higher than those reporting no adversities (44% of the sample).

While risk for early menarche appears to increase with a greater number of childhood adversities, the variable for the count of adversities (Model 1 in Table 3) imposes an assumption that each adversity is associated with an identical increase in the risk for early menarche. The 26% increase found with Model 1 is an average across all the adversities. Figure 1, in contrast, does not make this assumption. This apparent contradiction demonstrates the difficulty in selecting between different models of the association between adversities and early menarche. The findings appear to go against a simple, linear relationship and suggest that a threshold model is a better fit to the overall pattern in the data. That is, risk for early menarche appears to increase above a certain number of adversities.

These analyses were also designed to identify contributions of specific childhood adversities on early menarche (Model 2 in Table 3); findings are consistent with the suggestion in the literature that childhood sexual abuse is associated with earlier onset of menarche. As observational studies have limited ability to distinguish between effects of specific adversities and more generalized effects of childhood environment in the context of highly clustered adversities, this study included a much broader range of childhood adversities than prior studies. Specifically, of the 11 adversities, childhood sexual abuse had the strongest association with early menarche when each adversity was examined in a separate model. In models adjusting for co-occurrence among the five adversities most strongly associated with early menarche, only childhood sexual abuse remained associated with early menarche at a statistically significant level. Absence of biological father during childhood, theorized to affect early menarche due to early triggering of sexual development, was not associated with early menarche in this study.

These results are consistent with prior studies in finding that stressors in childhood may have biological influences on endocrine development resulting in early menarche, a key developmental milestone with important social and biological consequences that extend across adulthood. However, the evidence regarding specific mechanisms is mixed. Theories related to paternal investment, suggested by Draper and Harpending [55] as well as Belsky [56], hypothesizing that the absence of the biological father during childhood would trigger early sexual development have received some support. However, in this study, absence of the biological father was not associated with early menarche. The current findings are congruent with those of Ellis [36,37], in that the impact of early father absence on early menarche does not exist independent of other moderating factors in the environment. At the same time, the finding that sexual abuse prior to menarche is the strongest predictor of early menarche may implicate a related pathway. Several previous studies have also found that childhood sexual abuse is associated with early menarche [19,28,30,31,57,58], including a recent study showing links between childhood sexual abuse and early menarche in a large sample of African-American women [29] as well as a study suggesting a dose-response relationship between severity of sexual abuse and early menarche [34]. None of these studies including the current study, however, has included information on timing of pre-menarchal development of secondary sexual characteristics, which may contribute to increased risk for sexual abuse. Similarly, while beyond the scope of this study, examination of the differential impact of childhood adversities such as absence of biological father at different developmental stages (i.e., early childhood versus early adolescence) would help elucidate the specific pathways for the influence of childhood adversities on pubertal development.

This study has several limitations. Most importantly, we rely on retrospective reporting of both childhood adversities and age of menarche. A large literature examining associations of recalled childhood adversities with health outcomes has proven valuable despite the known limitation of retrospective assessment [59]. However, it is possible that certain adversities such as sexual abuse will be recalled more readily than others. Previous studies on recalled age of menarche found participant recall to be accurate within one year in 90% of cases between the teenage years and 30 years after the event [60,61]. As some of the study participants were in their 50s and 60s, it is possible that there is more recall bias with underreporting of childhood adversities and inaccurate timing of menarche among the older birth cohorts. In addition, data on BMI were available only from the time of interview as an adult, not from the period preceding menarche. This limitation is partially addressed by including participants’ BMI at the time of the interview in final analyses, as adult obesity and childhood obesity are highly correlated [62,63]. However, the self-reported adult height and weight to calculate BMI add an additional potential inaccuracy to these data (i.e., BMI is likely to be underestimated). Finally, information on the exact timing was not available for all adversities considered in the analysis, in particular for absence of biological father. It is possible in these cases that the adversity occurred after menarche and was misclassified in this study. Definitions have been constructed in these cases to minimize the possible impact of misclassification. It is unlikely that the remaining misclassification would result in bias in any particular direction.

Interpretation of the findings should also consider the absence of information on maternal age at menarche in this study and in most other studies of this topic. As noted by Moffitt and colleagues [24], early menarche may lead to early sexual debut and early marriage. Given that early age at marriage is a strong predictor of divorce, children of mothers with early age at marriage may be more likely to grow up in disadvantaged settings, in which they are at risk for a wide variety of childhood adversities. In this scenario, genetic transmission of age at menarche may underlie the observed associations between childhood adversities and early menarche. A study by Wise and colleagues included statistical control for maternal age at marriage, which would adjust in part for this pathway [29]. In that study, sexual abuse and physical abuse remained significantly associated with early menarche, although with associations of lesser magnitude than identified in this study.

## Conclusions

These findings are consistent with the theory that adverse family environments have physiologic effects on endocrine development and, through these effects, may impact mental and physical health outcomes in adulthood. Childhood sexual abuse is the adversity most strongly associated with early menarche. However, because of the complex way that childhood adversities cluster within families, the more generalized influence of highly dysfunctional family environments cannot be ruled out. More detailed studies that address gene-environment correlations as well as the possibility of confounding by pre-menarchal development are needed to advance towards more solid evidence that the associations observed here reflect a distinct bio-social etiology.

## Abbreviations

NCS-R: National Comorbidity Survey Replication.

## Competing interests

The authors declared that they have no competing interests.

## Authors’ contributions

KH conceptualized the study, conducted the literature review, participated in the design and analyses, drafted the initial manuscript, and approved the final manuscript as submitted. HLM participated in review of the analyses, critically reviewed and revised the manuscript, and approved the final manuscript as submitted. EM conceptualized and designed the study, reviewed and revised the manuscript, and approved the final manuscript as submitted. DS assisted in conceptualizing and designing the study, reviewed and revised the manuscript, and approved the final manuscript as submitted. NS participated in the design of the study, carried out the analyses, reviewed and revised the manuscript, and approved the final manuscript as submitted. JAB designed the study, directed the analyses, reviewed and revised the manuscript, and approved the final manuscript as submitted. All authors read and approved the final manuscript.
